# Multiple approaches to meso- and macroplastics and the food habitat of the green turtle, *Chelonia mydas*, in the Ogasawara Islands, Japan

**DOI:** 10.7717/peerj.20425

**Published:** 2026-01-02

**Authors:** Tenzo Fujitani, Shunji Ena, Touma Hosoya, Seongwon Lee, Miyuki Nishijima, Akira Iguchi, Haruka Nakano, Nozomu Iwasaki

**Affiliations:** 1 Islands Care, Ogasawara, Tokyo, Japan; 2 Faculty of Geo-Environmental Science, Rissho University, Kumagaya, Saitama, Japan; 3 Nikken Total Sourcing Inc., Saitama, Japan; 4 Gunmaken Shinkumi Bank, Gunma, Japan; 5 Geological Survey of Japan, National Institute of Advanced Industrial Science and Technology (AIST), Tsukuba, Ibaraki, Japan; 6 Center for Ocean Plastic Studies (COPS), Research Institute for Applied Mechanics (RIAM), Kyusyu University, Bangkok, Thailand

**Keywords:** Green turtle, *Chelonia mydas*, Mesoplastics, Macroplastics, Food habitat, Western North Pacific

## Abstract

This study integrated genetic, isotopic, and plastic analyses to investigate the diet and plastic ingestion of adult green turtles living in the waters around the Ogasawara Islands. Micro-, meso-, and macroplastics were found in the gut contents of 7 of the 10 individuals examined. A total of 92 meso- and macroplastics were found in six individuals, with an average of 9.2 ± 11.48 items/individual (range: 0–31, *n* = 10). The average total weight of these items was 15.28 ± 24.76 g (range: 0–70.55, *n* = 10), and their average percentage of the turtles’ body weight was 0.014 ± 0.021% (range: 0–0.064, *n* = 8). At the time of capture, the turtles’ main food was macroalgae, and the feeding grounds were estimated from the DNA analysis to be three locations where the seaweeds *Ectocarpus crouaniorum, Sargassum muticum*, and *Lobophora* sp. predominate. Stable isotope ratios of carbon and nitrogen in muscle tissue and DNA analysis suggested that the captured individuals may have fed on drifting seaweed and gelatinous plankton on their way south from the Pacific coasts of Japan to the Ogasawara Islands, during which time it can be concluded from the green turtle’s feeding habits that they may have ingested meso- and macroplastics found among large and drifting seaweeds, mistaking them for gelatinous plankton such as jellyfish and salpas. The ingested plastics were estimated to have originated from a larger area than the turtles’ migratory range, indicating that this problem may stem from transboundary pollution.

## Introduction

Since the development of modern plastics in 1907 and their mass production from the 1940s, large quantities of plastic waste have been released into the global environment, accumulating due to their durability (Thompson et al., 2009; Cole et al., 2011). The global plastic waste discharge was estimated in 2020 to be 52.1 (range: 48.3–56.3) million metric tons (Mt) per year, and 43% of this is at risk of being transported *via* land to the aquatic environment (Cottom, Cook & Velis, 2024). It is difficult to accurately gauge the quantity, but 275 million Mt of plastic waste is reported to have been generated in 192 coastal countries in 2010, and it is estimated that 4.8 to 12.7 million Mt of this flowed from land into the ocean (Jambeck et al., 2015). Moreover, plastic falls into the ocean from the atmosphere where it is suspended, and it is also brought there by human activities such as fishing and marine transport (Hardesty et al., 2017). Plastics reaching the ocean, particularly microplastics smaller than 5 mm, are subsequently ingested by marine organisms and enter the food chain (GESAMP, 2016; Lusher, Hollman & Mendoza-Hill, 2017). Experimental studies on marine organisms in low trophic levels indicate that microplastics may induce a sub-lethal response in them, negatively affecting their health, feeding, growth, and survival (GESAMP, 2016). Furthermore, it has been pointed out that some plastics remain in the marine environment without entering the food chain and that high-density plastics accumulate in sediment where, although they are unlikely to interact with biota, they may release toxic chemicals and plastic-associated contaminants or carry invasive alien species and microbial pathogens into the environment (De-la-Torre et al., 2021).

Plastic has been found in the bodies of many types of marine life, from pelagic fishes to whales and even zooplankton, as well as in deep-sea amphipods that inhabit depths of 7,000 to 10,890 m (Desforges, Galbraith & Ross, 2015; Lusher et al., 2015; Tanaka & Takada, 2016; Jamieson et al., 2019; Pereira et al., 2020). Most of the plastics found in marine organisms are microplastics (<5 mm). In the case of sea turtles, however, in addition to ingesting microplastics, it has been reported that they feed on the larger mesoplastics (5 mm to <2.5 cm) and macroplastics (2.5 cm to <1 m) (Schuyler et al., 2012; Hoarau et al., 2014; Duncan et al., 2019a, 2019b; Fukuoka et al., 2022; Kimura et al., 2024). In 2022, for example, a large clear plastic sheet (106 cm × 118 cm) was found in a dead stranded leatherback turtle, *Dermochelys coriacea*, in the waters off Japan (Deguchi et al., 2025). It has been suggested that the plastic ingested by sea turtles can be traced back to two sources: the non-selective ingestion of plastic mixed in with their normal food, such as algae, and the selective ingestion of plastic bags resembling one of their normal foods, jellyfish (Di Beneditto & Awabdi, 2014; Hoarau et al., 2014; Schuyler et al., 2014; Duncan et al., 2019a; Alfonso et al., 2024; Jandang et al., 2024).

The green turtle, one of five species of sea turtle distributed in the waters around Japan, nests in the Ogasawara Islands (Uchida & Nishiwaki, 1982), a chain of oceanic islands located approximately 1,000 km south of Tokyo off Honshu, the main island of Japan. Green turtles migrate from the Pacific coasts of Honshu to the Ogasawara Islands from March to May for mating and from May to August for nesting (Kamezaki & Matsui, 1977; Ogasawara Marine Center, 1990; Tachikawa & Sasaki, 1990; Tachikawa, 1991; Yamaguchi, Suganuma & Narushima, 2005). On their way to and from the Ogasawara Islands, the turtles may therefore ingest and accumulate plastic debris distributed in waters from the coast to the open ocean in various forms (suspended in water, deposited on sediment, mixed among seaweeds, *etc*.). This study extends prior research (*e.g*., Fukuoka et al., 2016) by integrating genetic, isotopic, and plastic analyses to investigate the factors contributing to plastic ingestion and estimate the origins of plastics ingested by this unique population of green turtles in the Ogasawara Islands.

## Materials and Methods

### Sampling

Sampling was conducted from 13 to 23 March 2021, at Hahajima Fishing Port, Hahajima Island, Ogasawara Islands, Japan (26°38′17.5″N, 142°9′33.2″E). Mesoplastics (5 mm to <2.5 cm) and macroplastics (2.5 cm to <1 m) (GESAMP, 2015) were collected from the large intestine of 10 adult green turtles (four females and six males, including three pairs for mating) caught by fishermen in the waters around Hahajima. The gut contents of the large intestine and tissue samples from the lining of the intestine and the rectus abdominis muscle were also collected (Table 1). The samples were stored at −18 °C immediately after sampling and at −30 °C after transportation to the laboratory.

**Table 1:** Sample information of green turtles, *Chelonia mydas*, collected from Hahajima I., Ogasawara Is., Japan.

Turtle number	Date of sampling	Sex	Body		Gut content wet weight (g)
			Length* (cm)	Wet weight (kg)	
1^a^	13 March 2021	M	160	–	498.3
2^a^	13 March 2021	F	165	168	538.5
3^b^	13 March 2021	M	154	105	396.9
4^b^	13 March 2021	F	162	154	330.1
5^c^	15 March 2021	M	132	–	483.7
6^c^	15 March 2021	F	124	157	419.8
7	20 March 2021	M	147	102	723.9
8	20 March 2021	M	133	103	498.5
9	20 March 2021	F	150	148	437.0
10	23 March 2021	M	135	111	535.6

**Notes:**

M, male; F, female.

^a–c^The same alphabet indicates a pair for mating.

* The length was measured from the tip of the beak to the tip of the tail.

The turtles were weighed and the length measured from the tip of the beak to the tip of the tail using large vernier calipers prior to sample collection. The sex and maturity of the captured individuals were determined by naked-eye observation of the gonads at the time of dissection. In three cases, a male and a female were captured together at the same time in the same location. Since mating behaviour of green turtles is observed in the waters around the Ogasawara Islands from February to June (Yamaguchi, Suganuma & Narushima, 2005), we concluded that these were mating pairs.

Permission to conduct sampling was granted in advance by the Ogasawara Hahajima Fisheries Cooperative. Green turtle fishery is licensed by the Governor of Tokyo, and the fishermen who provided the samples had obtained that licence. The green turtles died at the time of capture and samples for this study were taken from the dead individuals.

### Gut contents

A portion of the contents of the large intestine (0.4–10.6% of the wet weight of the collected sample) of each turtle, numbered from 1 to 10, was collected and weighed. Using a stereo microscope, each sample was then identified and separated into three categories: seaweeds, animal fragments, and non-organic matter such as microplastics. The wet weight of each category was measured and its percentage of the wet weight of the sample was determined. For seaweeds, their percentage of the wet weight of the sample excluding non-organic matter was also calculated.

### DNA analysis

The contents of the large intestine of turtles no. 1 to 10 were analysed. One sample each was taken from turtles no. 1 to 9, and two samples were taken from turtle no. 10 as a methodological control to validate consistency. DNA was extracted from approximately 2 g of each sample using a DNeasy PowerMax Soil kit (manufactured by Qiagen, Hilden, Germany), following the kit’s attached instructions. To detect eukaryotes in the samples using next generation sequencing (NGS), a PCR was performed targeting the 18S ribosomal RNA gene (18S rRNA) of eukaryotes. The PCR targeted the V1–V2 region of the 18S rRNA gene, the primers including the overhang adapter sequence (part in parentheses) for MiSeq analysis: F04:5′-[ACACTCTTTCCCTACACGACGCTCTTCCGATCT]GCTTGTCTCAAAGATTAAGCC-3′ (Blaxter et al., 1988); R22mod: 5′-[GTGACTGGAGTTCAGACGTGTGCTCTTCCGATCT]CCTGCTGCCTTCCTTRGA-3′ (Sinniger et al., 2016) were used. All PCR reactions included a negative control using ultrapure water instead of template DNA.

To eliminate bias caused by PCR amplification, PCR reactions were performed using three tubes per sample and combining the three tubes into one after confirming the presence or absence of amplified products by agarose gel electrophoresis. The obtained PCR amplified products were purified using magnetic beads (Agencourt AMPure XP Kit: Beckman Coulter, Brea, CA, USA), and second-round PCR (indexing PCR) was performed to add index sequences to distinguish each sample during MiSeq analysis. After purification of the indexing PCR products using magnetic beads, a next-generation sequencer (MiSeq, Illumina, Los Angeles, CA, USA) with MiSeq Reagent Kit V3 (Illumina, Los Angeles, CA, USA) with paired-end sequencing (2 × 300 bp) was used to analyse the samples. The obtained sequences formatted as FASTQ files were analysed using QIIME 2 (v2021.02.0, Bolyen et al., 2019), and quality control including quality filtering, denoising, correcting reading errors, merging paired-end reads, and removal of non-biological sequences (including chimera) was performed. Amplicon sequence variants (ASVs) were obtained by DADA2 (Callahan et al., 2016), and a count table of representative sequences was generated with dereplication of the ASVs. Taxonomic assignment to ASVs was referred by the database SILVA 138 (SILVA rRNA Database Project, 2019).

Additionally, for each representative sequence, homology searches were conducted with the National Center for Biotechnology Information (NCBI) nucleotide sequence database using BLAST+ (Camacho et al., 2009) and the taxonomic group was estimated. Only sequences that were assigned to each taxon with a longer length of at least 100 bp and a similarity rate of at least 90% were used in the subsequent statistical analysis. Furthermore, of the total number of eukaryotic reads detected, those derived from reptiles, including the green turtle, and those of *Blastocystis* (Stramenopiles), which are thought to be intestinal parasites, were excluded. The resulting FASTQ files were deposited in the DNA Data Bank of Japan (DDBJ) (accession: PRJDB20659).

Based on the identification results, the life form (plankton, benthos, nekton, and phytal) of each taxon in the ocean environment was estimated (Mukai, 1971; Boero et al., 2008; Sommer, 2023). The similarity of seaweed composition between the males and females of the mating pairs was calculated using Whittaker’s percentage similarity index (PS) with the following formula (Whittaker, 1952):

$PS = \sum \min (Mi, Fi)$

where *Mi* is the percentage of seaweed *(i)* in the male and *Fi* is the percentage of seaweed *(i)* in the female.

### Carbon and nitrogen stable isotope ratio analysis

The bulk of the gut contents and the rectus abdominis of each green turtle were analysed. For turtle no. 10, the lining of the intestine and seaweed taken from the bulk of the gut contents were also analysed. The gut contents were first treated with 1N hydrochloric acid to remove inorganic carbon. The pretreated gut contents, the rectus abdominis and the lining of the intestine were defatted with a methanol:chloroform mixture (volume ratio 1:2) and washed with Milli-Q water. All samples were freeze-dried.

Stable isotope ratio mass spectrometry (Flash 2000-ConFlo VI-DELTA V PLUS; Thermo Fisher Scientific, Waltham, MA, USA) was used for the analysis. Each biological sample was analysed 3 to 4 times to reduce variation in the analysis values. The standard uncertainty was (*n* = 10) is ±0.10‰ for δ^13^C and ±0.15‰ for δ^15^N. The average and standard deviation of each sample was reported as the analysis value. The analysis value for the gut contents of turtle no. 6 differed from the values for the other individuals, so another sample was taken and analysed, and the average of the two samples was used. The isotope ratios were expressed in per mil deviations from a standard as defined by the following equation:

${\rm \delta^{13}C\; or\; \delta^{15}N = [(R_{sample} - R_{standard}) / R_{standard}] \times 1000}$

where R denotes ^13^C/^12^C or ^15^N/^14^N. Vienna Pee Dee Belemnite (VPDB) (IAEA-LEVEC, IAEA-CH-6, NBS-18) and atmospheric nitrogen (IAEA-N-1, IAEA N-2, USGS 26) were used as the standard for δ^13^C and δ^15^N, respectively.

### Plastics

The plastic collected from the large intestine was placed in a 10% potassium hydroxide solution and left at 50 °C for 2 days. It was then transferred to a 30% hydrogen peroxide solution and left at 50 °C for 3 days. After that, it was rinsed with distilled water and dried at room temperature (GESAMP, 2016). The weight and length of the plastic items were then measured. The length was measured along the shortest and longest sides, and these were multiplied to give an index of size. The area of larger, irregularly shaped items with a long diametre of 100 mm or more was measured using the image processing package Fiji (Schindelin et al., 2012). Plastic size is defined as microplastics measuring less than 5 mm in diameter, mesoplastics from 5 mm to less than 2.5 cm, macroplastics from 2.5 cm to less than 1 m, and megaplastics 1 m or more (GESAMP, 2016). This definition was difficult to apply to the many irregularly shaped items ingested by the green turtles in this study, so we used the area of the plastic as an indicator of size. The weight and percentage of plastic per body weight of the green turtles were determined using the following equations:

$\rm {Weight\; of\; plastic\; per\; body\; weight = weight\; \; plastics\; (g) / weight\; of\; green\; turtle\; (kg).}$

Percentage of plastic per body weight = weight of plastics (g)/weight of green turtle (g) × 100.

The plastic items were classified into three categories: film (thin and soft), fragments, and lines (Lusher, Hollman & Mendoza-Hill, 2017). The colour categories were clear, translucent, white, black, and coloured (red, yellow, green, blue, purple).

The infrared absorption spectra of the plastic samples were measured using an FT/IR-4600 infrared spectrophotometer (JASCO Corporation, Tokyo, Japan) equipped with an ATR accessory using a diamond prism. The spectral data of the samples were compared with the library data stored in KnowItAll (Bio-Rad Laboratories Inc., Hercules, CA, USA). If the Hit Quality Index (HQI) for the material indicated in the library search results was greater than 70, it was judged that the material of the sample matched the material indicated in the search results. A total of 88 samples were analysed. Seven of the 88 analysed samples were estimated to be cellulose, not plastics, so these were excluded from the analysis. The HQI exceeded 70 in 44 of the 81 plastic samples. Finally, microplastics in the gut contents were counted and the presence of microplastics in DNA samples was recorded.

## Results

### Plastics

The mean body length of the green turtles we studied was 143.5 ± 11.9 (*n* = 6) for the males and 150.3 ± 18.7 (*n* = 4) for the females, and there was no significant difference between them (*t*-test, *t*(6) = 0.71, *p* = 0.50). The mean body weight was 105.3 ± 4.0 (*n* = 4) for the males and 156.8 ± 8.4 (*n* = 4) for the females, and the difference between them was significant (*t*-test, *t*(6) = 11.07, *p* < 0.001) (Table 1). A total of 92 meso- and macroplastics with a length of more than 5 mm were found in 6 of the 10 green turtles, with an average of 9.2 ± 11.48 (range: 0–31, *n* = 10) items/individual. Spearman’s correlation coefficient was used to determine whether there was a correlation between body length and the number of plastic items. The result showed no significant correlation (*r* = 0.14, *p* = 0.71). A total of 13.0 ± 10.23 items/individual (range: 1–26, *n* = 4) of meso- and macroplastics were found in the large intestine of females and 6.7 ± 13.44 items/individual (range: 0–31, *n* = 6) in males. A Mann-Whitney U test was performed to determine whether the number of plastic items differed significantly between genders. The result showed no significant gender difference (*U* = 19, *p* = 0.12). Microplastics were also detected in the gut contents and samples for DNA, bringing the total number of individuals found to have plastics to seven (Table 2). Three individuals, all male, had no meso- or macroplastics in the large intestine nor any microplastics in the gut contents or samples for DNA. The average total weight of meso- and macroplastics for each individual was 15.28 ± 24.76 g (range: 0–70.55, *n* = 10). The average weight of plastic per body weight of the turtles was 0.14 ± 0.23 g plastic/kg turtle (*n* = 8), with a maximum of 0.64 g plastic/kg turtle. The average percentage of body weight made up of plastic was 0.014 ± 0.021% (range: 0–0.064, *n* = 8) (Table 2). Turtle no. 10 had the greatest number and volume of plastic items and the highest percentage of plastic weight to body weight at 0.064%. On naked-eye observation, the inner wall of the intestine of this turtle was mottled and blackened.

**Table 2:** Plastics in green turtles, *Chelonia mydas*, collected from Hahajima I., Ogasawara Is., Japan.

Turtle number	Sex	Plastics in gut		Plastic weight per body weight (g/kg)	Percentage of plastics in body weight (%)	Presence of microplastics	
		Number	Total weight (g)			In gut content sample	In DNA sample
1^a^	M	0	0	–	–		
2^a^	F	12	25.25	0.15	0.015	^	^
3^b^	M	0	0	0	0		
4^b^	F	26	46.83	0.30	0.030	^	
5^c^	M	9	4.70	–	–	^	
6^c^	F	1	1.00	0.01	0.001	^	
7	M	0	0	0	0		
8	M	0	0	0	0		^
9	F	13	4.45	0.03	0.003	^	
10	M	31	70.55	0.64	0.064	^	^
Average		9.2	15.28	0.14	0.014		
Frequency of occurence (%)		60 (70*)				60	30

**Notes:**

M, male; F, female.

^a–c^The same letter indicates a pair for mating.

* Including the presence of plastics in both gut contents and DNA samples.

–Body weight data not available.

**Figure 1:**
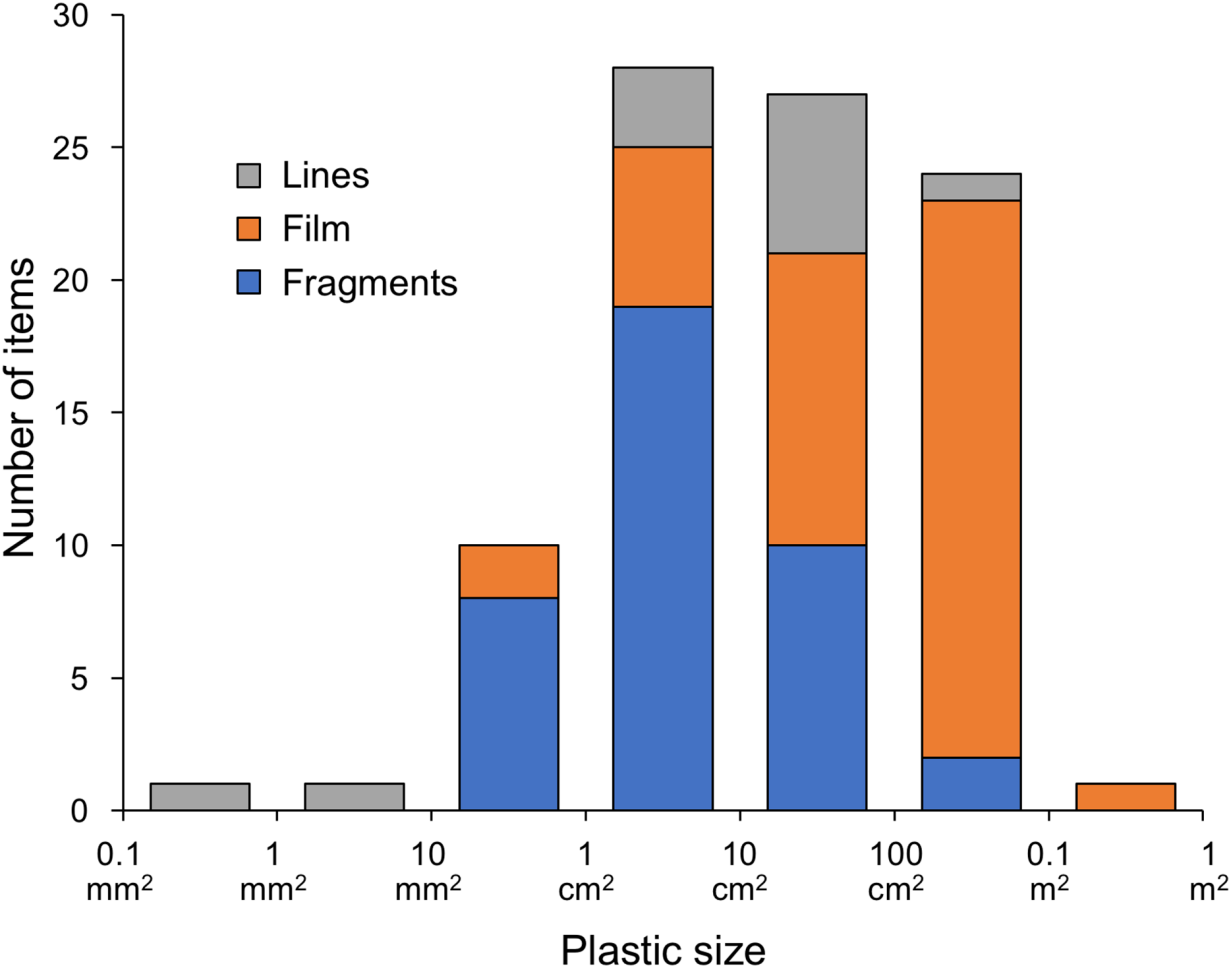
Proportion of shapes for each size category of ingested plastics collected from the large intestine of adult green turtles, *Chelonia mydas*, in Hahajima I., Ogasawara Islands, Japan.

**Figure 2:**
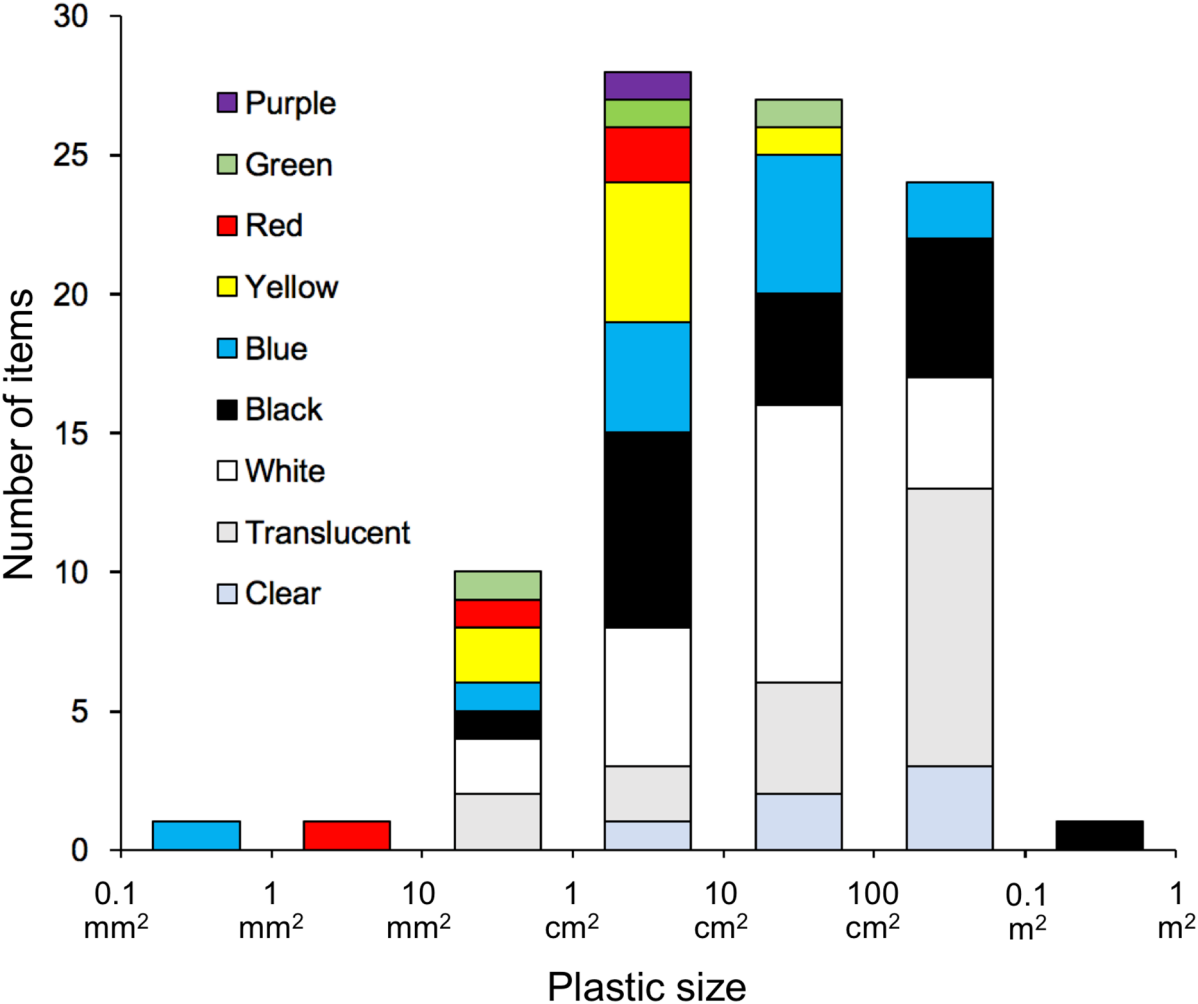
Proportion of colours for each size category of ingested plastics collected from the large intestine of adult green turtles, *Chelonia mydas*, in Hahajima I., Ogasawara Islands, Japan.

**Figure 3:**
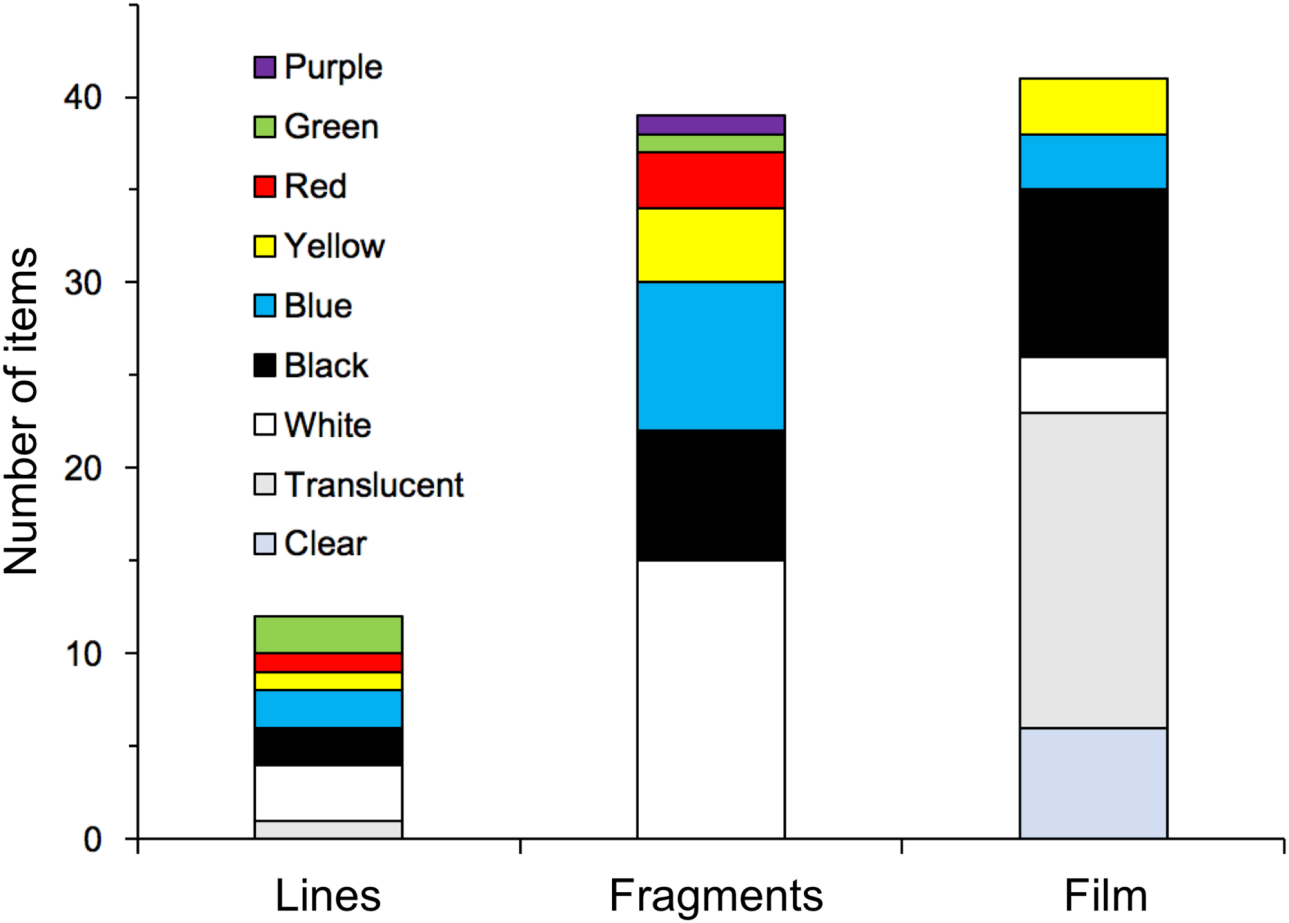
Proportion of colours for each shape category of ingested plastics collected from the large intestine of adult green turtles, *Chelonia mydas*, in Hahajima I., Ogasawara Islands, Japan.

**Figure 4:**
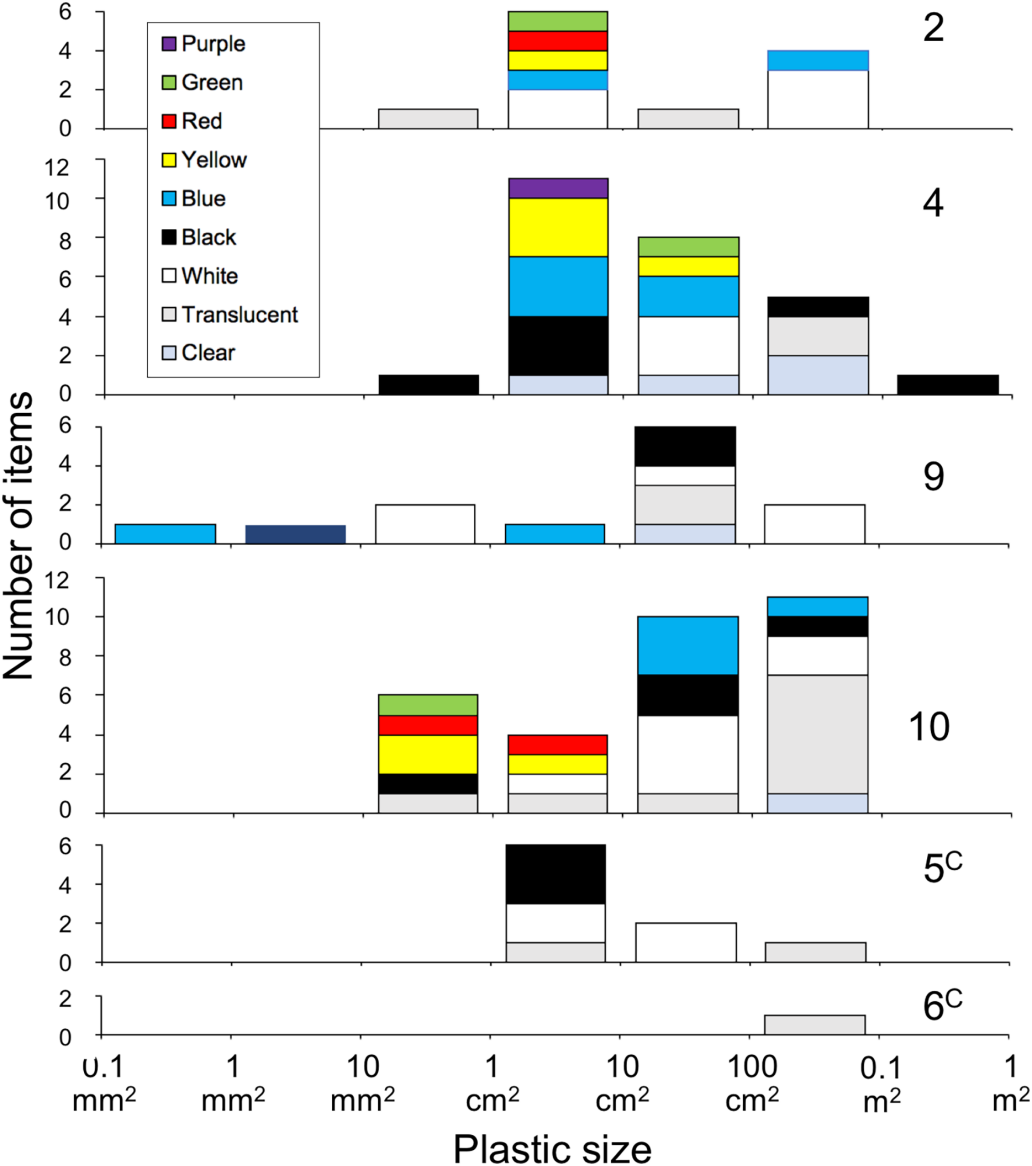
Frequency distribution of the size of ingested plastics for each adult green turtle, *Chelonia mydas*, in Hahajima I., Ogasawara Islands, Japan. Turtles no. 6 to 2 are arranged in ascending order of body length. ^c^The same letter indicates a pair for mating.

As for the morphology of the plastics, 56.5% of the items measured 10 cm^2^–1 m^2^, corresponding to macroplastic, and 41.3% measured 10 mm^2^–10 cm^2^, corresponding to mesoplastic, with film, fragments, and lines accounting for 44.6%, 42.4%, and 13.0%, respectively. A large proportion of the 10 mm^2^–10 cm^2^ size category consisted of fragments, accounting for 71.1%. The proportion of film increased with the size of the plastic, accounting for 88.0% of the 100 cm^2^–1 m^2^ size category (Fig. 1). The colours of the plastics were classified as follows: clear/translucent accounted for 26.1%, white for 22.8%, black for 19.6%, and coloured for 29.0%. Excluding the largest size, the proportion of clear/translucent and white increased with the size of the plastic (Fig. 2), and a significant relationship was found between them (χ^2^ test, χ^2^ = 225.04, *p* < 0.001). The proportion of clear/translucent and white was 63.4% for film, which was slightly higher than the 33.3% and 38.5% for lines and fragments, respectively (Fig. 3). The results of FT-IR analysis showed that polyethylene was the most common plastic material, accounting for 79.5% of the total. This was followed by polypropylene at 15.9%, and the remainder was polystyrene at 4.5%. The frequency distribution of the size of ingested plastics for each green turtle is shown in Fig. 4. There were no significant differences in the size distribution of plastics among individuals (one-way ANOVA, *F*(5, 86) = 2.14, *p* = 0.07), and there was no tendency for larger individuals to ingest larger plastics.

**Figure 5:**
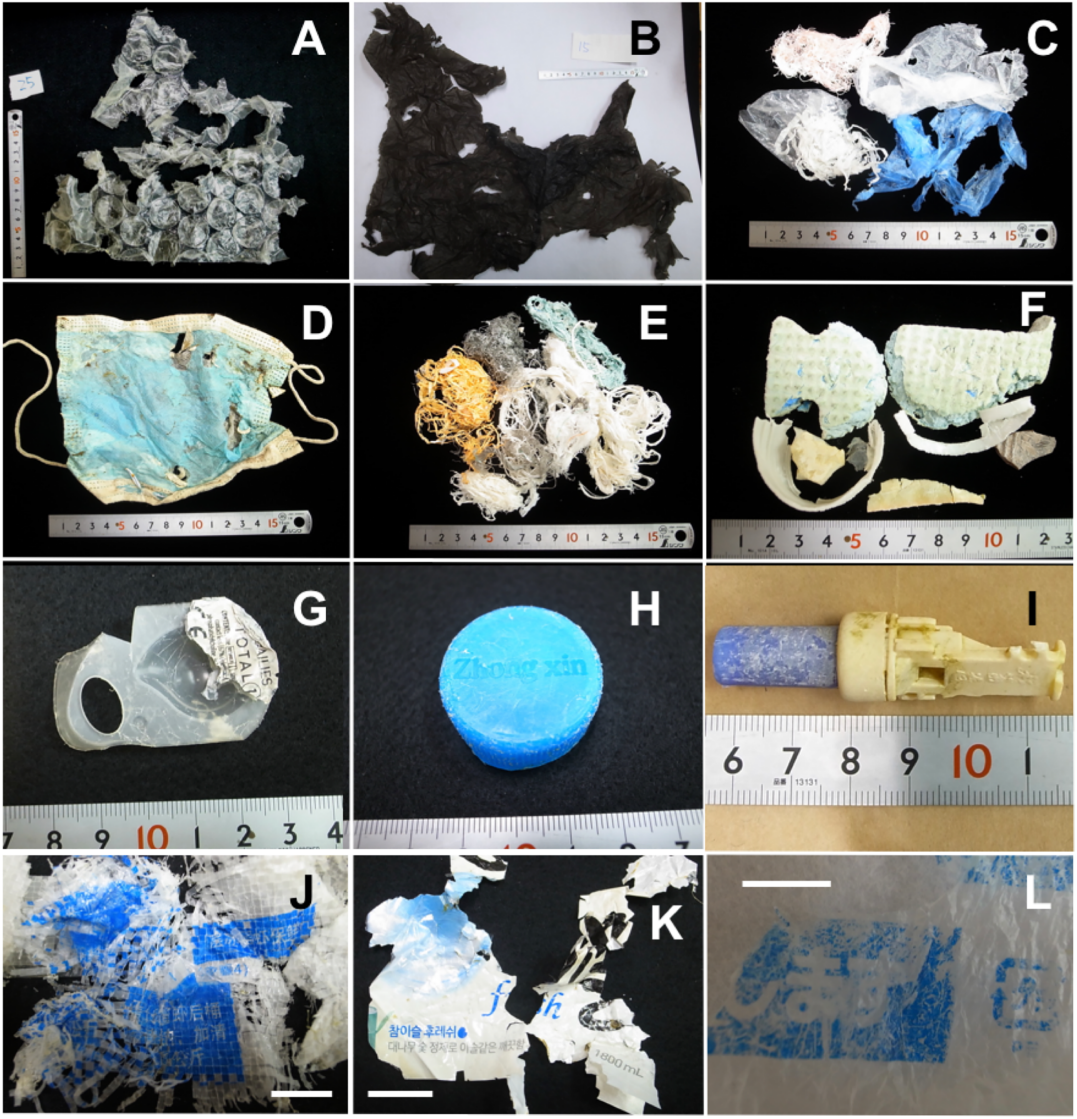
Plastics collected from the large intestine of adult green turtles, *Chelonia mydas*, in Hahajima I., Ogasawara Islands, Japan. (A) Air cushion film in turtle no. 4, polyhydrocarbon (Petrolite c-4040). (B) Black film in turtle no. 4, polyhydrocarbon (Petrolite c-4040). (C) White lines and translucent film in turtle no. 10. (D) Medical face mask in turtle no. 10. (E) Clumps of lines in turtle no. 10. (F) Unidentified items in turtle no. 10. (G) Contact lens blister pack in turtle no. 9. (H) PET bottle cap inscribed with Pinyin in turtle no. 4, polyethene (Petrothene HD 5002). (I) Whistle with Chinese characters in turtle no. 4, polyethene (Vestolen P2300). (J) Sheet with simplified Chinese characters in turtle no. 2; scale bar indicates 2 cm. (K) PET bottle label with Korean letters in turtle no. 4; scale bar indicates 2 cm. (L) Sheet with Japanese characters in turtle no. 4; scale bar indicates 1 cm.

Photographs of plastics retrieved from the large intestine are shown in Fig. 5. Some of these items are clearly recognisable: *e.g*., a medical face mask (Fig. 5D), used extensively around the world during the COVID-19 pandemic; a contact lens blister pack (Fig. 5G); a PET bottle cap (Fig. 5H); and a whistle (Fig. 5I). The pet bottle cap has ‘Zhong xin’ written on it in Pinyin romanization, and the whistle has Chinese characters. Other items have simplified Chinese characters (Fig. 5J), Korean letters (Hangul) (Fig. 5K), and Japanese characters (kana and hiragana) (Fig. 5L) printed on them.

### Gut contents

The gut contents were mostly seaweeds, averaging 84.42% (range: 36.11–100%, *n* = 10). When the occurrence of seaweeds was calculated excluding non-organic matter that provides no nutrition for sea turtles, the average was 97.27% (range: 75.95–100%, *n* = 10). Animal fragments such as crustacean exoskeletons and shells were found in two individuals, accounting for 23.53% of the gut contents in turtle no. 7 (Table 3). Turtles no. 2, 4, and 10 had a high proportion of non-organic matter, accounting for 25.3%, 63.9%, and 39.2%, respectively. The non-organic matter consisted mainly of plastics, which were black, yellow, brown, and pink in colour.

**Table 3:** Gut contents of green turtles, *Chelonia mydas*, collected from Hahajima I., Ogasawara Is., Japan.

Turtle number	Wet weight (g)					Percentage				Percentage of seaweed in gut contents excluding non-organic matter
	Seaweed	Animal fragments	Non-organic matter	Unknown	Total	Seaweed	Animal fragments	Non-organic matter	Unknown	
1^a^	2.07	0.05	0.00	0.02	2.14	96.73	2.34	0.00	0.93	96.73
2^a^	9.83	0.00	3.32	0.00	13.15	74.75	0.00	25.25	0.00	100.00
3^b^	22.85	0.00	0.00	0.00	22.85	100.00	0.00	0.00	0.00	100.00
4^b^	3.30	0.00	5.84	0.00	9.14	36.11	0.00	63.89	0.00	100.00
5^c^	10.14	0.00	0.02	0.00	10.16	99.80	0.00	0.20	0.00	100.00
6^c^	44.64	0.00	0.00	0.00	44.64	100.00	0.00	0.00	0.00	100.00
7	11.62	3.60	0.00	0.08	15.30	75.95	23.53	0.00	0.52	75.95
8	10.28	0.00	0.00	0.00	10.28	100.00	0.00	0.00	0.00	100.00
9	11.54	0.00	0.00	0.00	11.54	100.00	0.00	0.00	0.00	100.00
10	5.50	0.00	3.54	0.00	9.04	60.84	0.00	39.16	0.00	100.00

**Note:**

^a–c^The same letter indicates a pair for mating.

### DNA analysis

**Figure 6:**
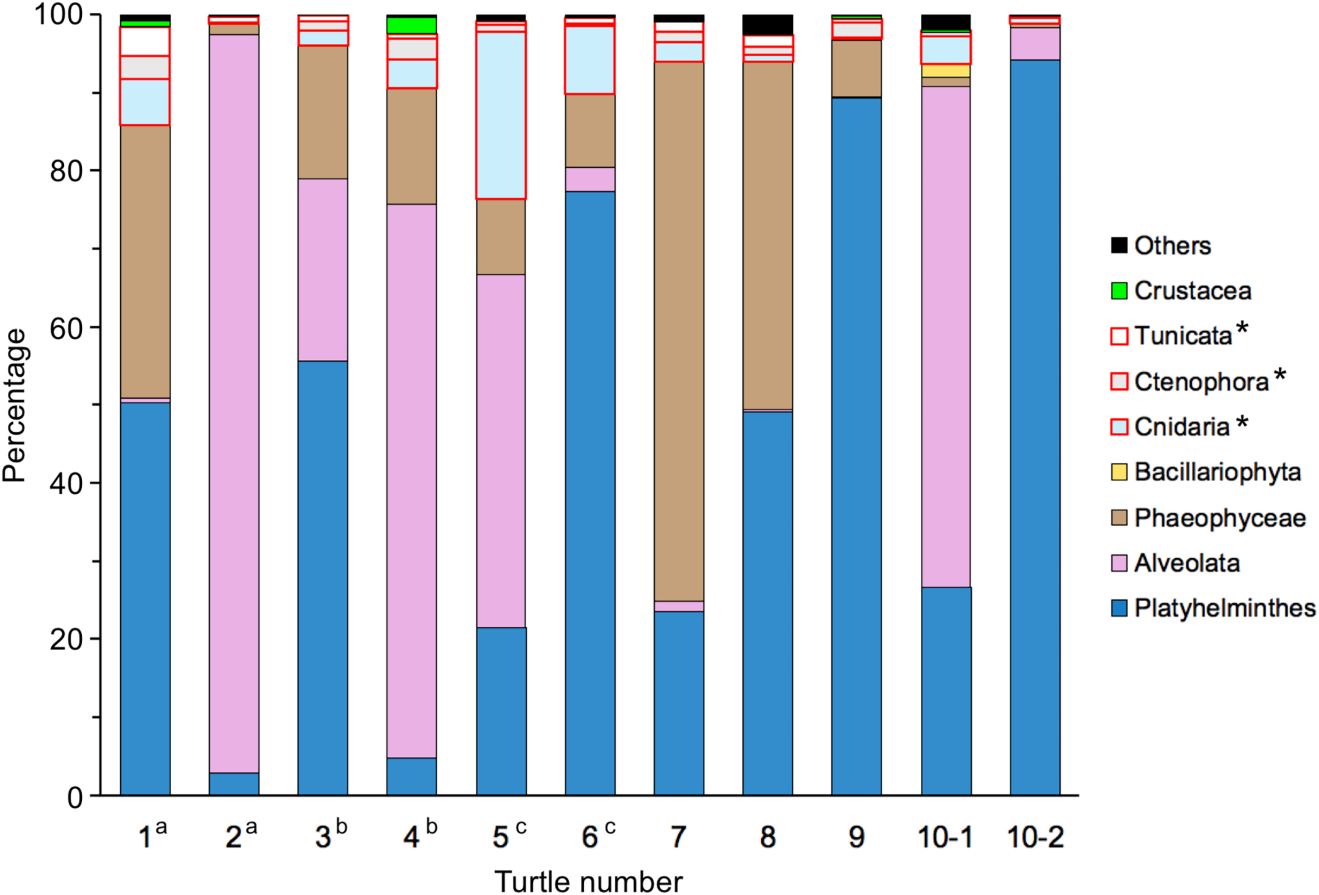
Composition of sequence reads for eukaryotes excluding sea turtles, parasites, and uncultured eukaryotes, identified from gut contents of adult green turtles, *Chelonia mydas*, in Hahajima I., Ogasawara Islands, Japan. ^a-c^ The same letter indicates a pair for mating. * Gelatinous plankton.

The sequences obtained using the next-generation sequencer were analysed using QIIME 2, and 339 representative sequences (ASVs) were obtained. Furthermore, when taxonomic groups were estimated using BLAST+, 326 ASVs were assigned to eukaryotes. Of the eukaryotic reads contained in the contents of the large intestine, the number of Platyhelminthes (flatworms), Alveolata (flagellates, dinoflagellates, *etc*.), and brown algae (Phaeophyceae) was high, accounting for an average of 44.99% (range: 2.80–94.21%, *n* = 11), 28.00% (range: 0.08–94.66%, *n* = 11), and 19.10% (range: 0.48–69.17%, *n* = 11), respectively (Fig. 6). The proportions of each taxon differed between individuals, and there were no common characteristics within the same sex. The composition of the two samples analysed for turtle no. 10 differed, with Alveolata being more abundant in sample 10-1 (64.12%) and Platyhelminthes in 10-2 (94.21%). There was a significant difference in composition between individuals (Friedman test, χ^2^ = 48.31, *p* < 0.001).

**Figure 7:**
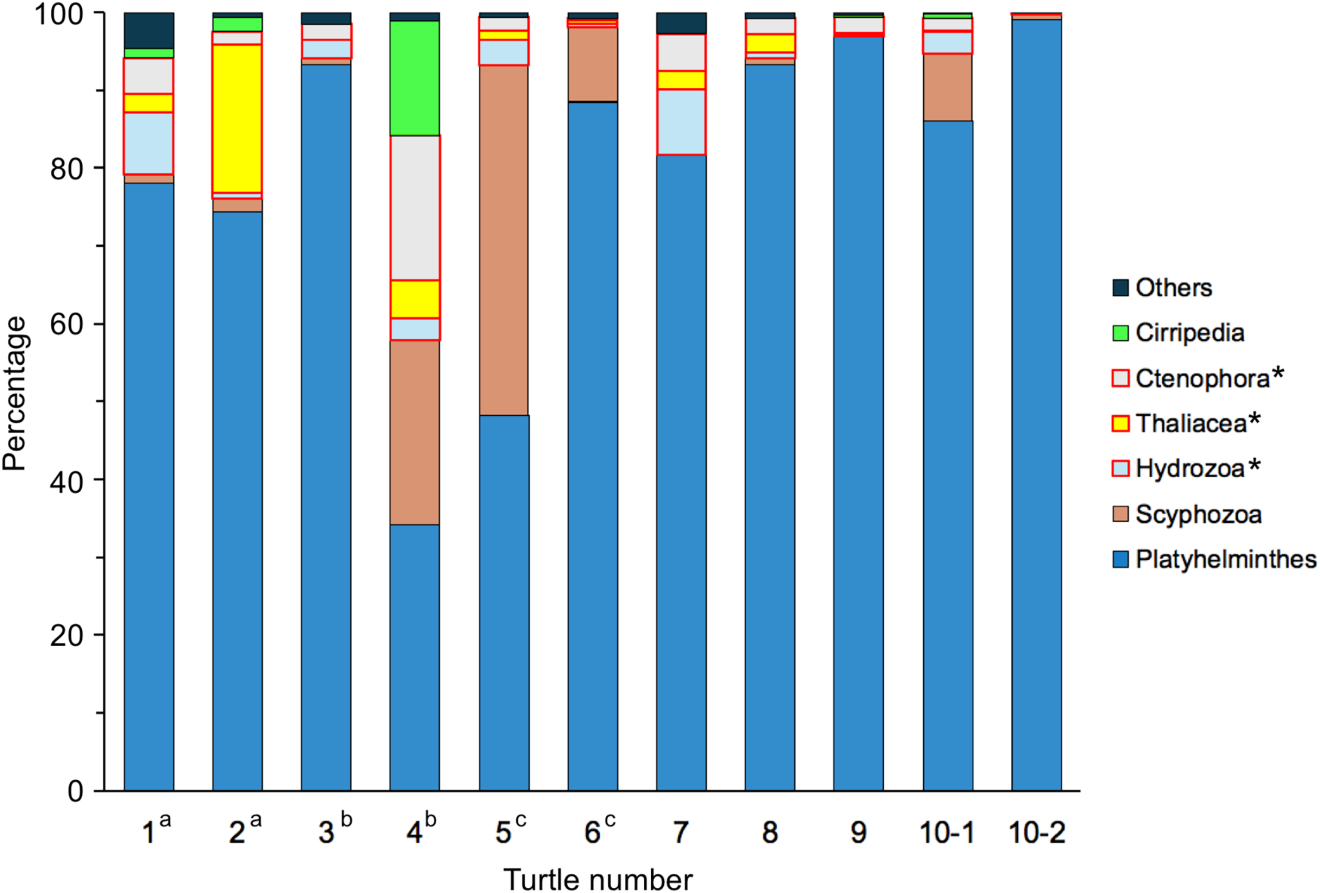
Composition of sequence reads for metazoans identified from gut contents of adult green turtles, *Chelonia mydas*, in Hahajima I., Ogasawara Islands, Japan. ^a–c^ The same letter indicates a pair for mating. * Gelatinous plankton.

The proportion of each taxon in the phylum of the metazoans was the highest for Platyhelminthes, which accounted for an average of 79.44% (Fig. 7). This taxon includes *Carettacola hawaiiensis* (Dailey, Fast & Balazs, 1992) and *Learedius learedi* (Kitayama et al., 2019; Morick et al., 2023), which was isolated from the hepatic blood vessels or the infected heart and blood vessels of a green turtle. Scyphozoa (true jellies) (8.35%) and Ctenophora (comb jellies) (3.59%) were also common, with Thaliacea (3.00%) and Hydrozoa (2.73%) bringing the proportion of gelatinous plankton to 17.67%. Looking at the life forms, benthic and/or phytal organisms accounted for an average of 80.47%, while planktonic accounted for 19.37% (Table 4). A Friedman test was performed to determine whether the proportion of each metazoan taxon differed among individuals. Sample 10-1 was used for turtle no. 10. The results showed significant differences (χ^2^ = 28.56, *p* < 0.001), indicating that each individual had ingested different proportions of metazoanic taxa.

**Table 4:** Percentage of the number of reads in life forms to the number of reads in metazoans in the gut contents of green turtles, *Chelonia mydas*, collected from Hahajima I., Ogasawara Is., Japan.

Life form	Taxon	Turtle number										Average
		1^a^	2^a^	3^b^	4^b^	5^c^	6^c^	7	8	9	10	
Benthos	Anthozoa, Nemertea, Decapoda, Echinodermata			0.22	0.19		0.02		0.10			0.05
Benthos/Phytal	Porifera, Platyhelminthes, Bryozoa, Gastropoda, Polychaeta, Entoprocta, Cirripedia, Ascidiacea	83.95	76.86	94.63	49.35	48.27	89.19	84.42	94.03	97.38	86.62	80.47
Plankton/Phytal	Copepoda				0.25		0.04				0.09	0.04
Plankton	Hydrozoa, Scyphozoa, Ctenophora, Branchiopoda, Thaliacea	16.05	23.14	5.15	50.22	51.15	10.74	15.58	5.88	2.58	13.23	19.37
Nekton	Osteichthyes					0.58				0.04	0.06	0.07

**Note:**

^a–c^ The same letter indicates a pair for mating.

**Figure 8:**
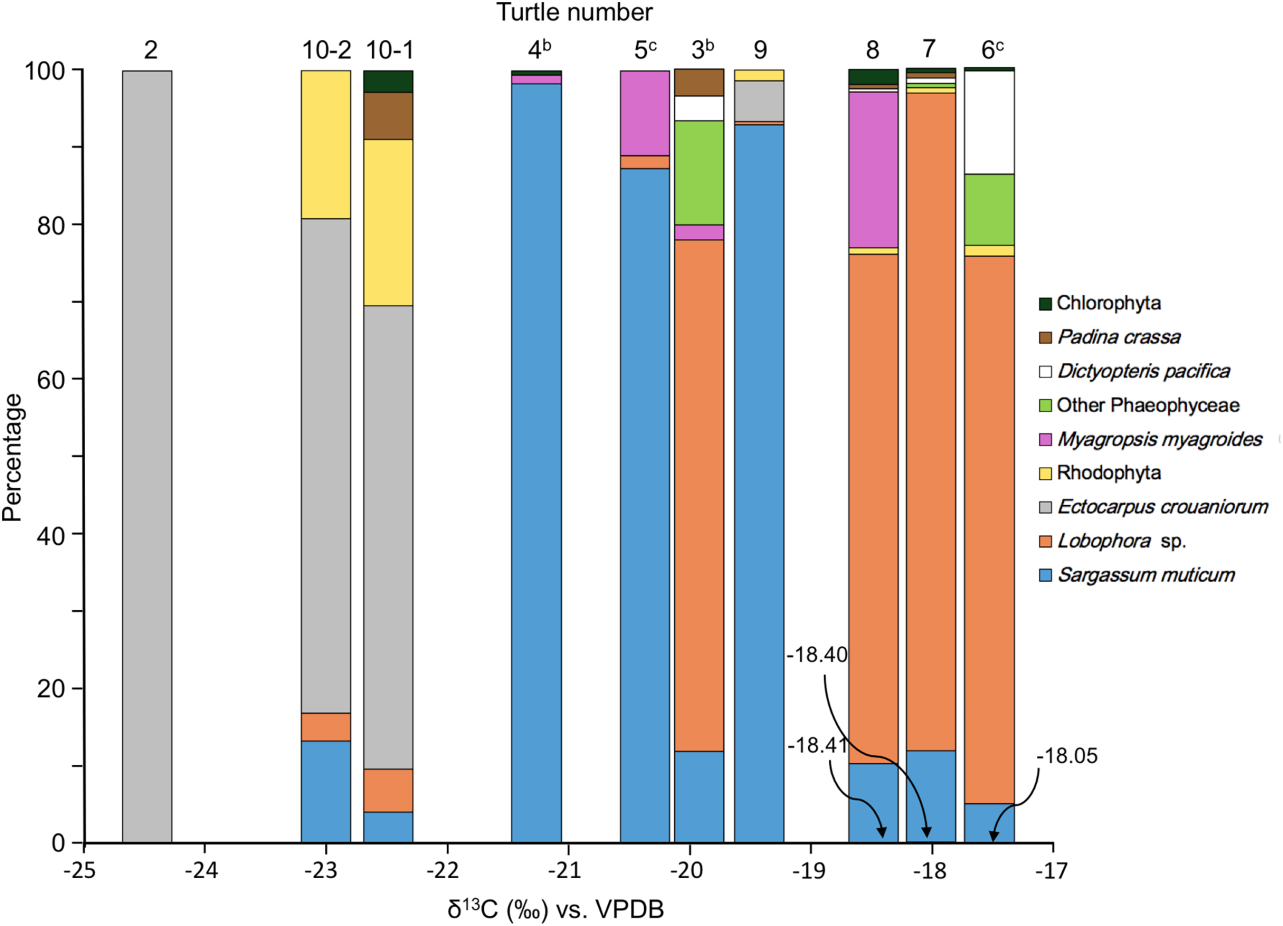
Composition of sequence reads for seaweeds in order of nitrogen stable isotope ratio, identified from gut contents of adult green turtles, *Chelonia mydas*, in Hahajima I., Ogasawara Islands, Japan. ^b–c^ The same letter indicates a pair for mating.

Of the gut contents identified as undigested prey, 97.27% were seaweeds. On the other hand, genetic analysis methods can detect eukaryotes if DNA remains even if it is not visible, so in terms of the number of reads, seaweeds (Chlorophyta, Rhodophyta, Phaeophyceae) accounted for 17.84% of the eukaryotes detected. Fifteen species of brown algae (Phaeophyceae) were identified, accounting for 95.38% of the seaweeds. Among these, *Surgassum muticum* (average 35.17%), *Lobophora* sp. (31.26%), and *Ectocarpus crouaniorum* (20.85%) were particularly common. There were significant differences between individuals in the species composition of seaweeds (Friedman test, χ^2^ = 17.44, *p* < 0.05). Turtles no. 1 and 10 were dominated by *Ectocarpus crouaniorum*, turtles no. 4, 5 and 9 by *S. muticum*, and turtles no. 3, 6, 7 and 8 by *Lobophora* sp. (Table 5, Fig. 8). Thus, the types and proportions of seaweed eaten by each individual differed. Furthermore, the seaweed composition in the pairs (turtles no. 1–2, 3–4, and 5–6) differed between the male and female, with a low percentage similarity for each pair of 0%, 13.0%, and 6.8%, respectively. As for the two samples (10-1 and 10-2) collected from the gut contents of turtle no. 10, the samples differed in terms of the composition of sequence reads for eukaryotes (Fig. 6), but there was little difference between them in the composition of sequence reads for seaweed (Fig. 8).

**Table 5:** Species composition (%) of seaweed based on the number of reads in the gut contents of green turtles, *Chelonia mydas*, collected from Hahajima I., Ogasawara Is., Japan.

Taxon		Turtle number											Average
		1^a^	2^a^	3^b^	4^b^	5^c^	6^c^	7	8	9	10	10*	
Rhodophyta		–	–	–	–	–	1.38	0.57	0.86	1.33	21.58	19.20	4.08
Chlorophyta		–	–	–	0.51	–	0.36	0.47	1.89	–	2.70	–	0.54
Phaeophyceae													
Dictyotaceae	*Dictyopteris pacifica*	–	–	3.16	–	–	13.41	0.63	0.33	–	–	–	1.59
	*Dictyopteris* *polypodioides*	3.08	–	–	–	–	–	–	–	–	–	–	0.28
	*Dictyota coriacea*	–	–	–	–	–	1.52	–	–	–	–	–	0.14
	*Distromium decumbens*	–	–	1.34	–	–	4.55	0.66	–	–	–	–	0.59
	*Homoeostrichus* *formosana*	–	–	–	–	–	0.85	–	–	–	–	–	0.08
	*Lobophora* sp. PALNG021	44.93	–	66.16	–	1.66	70.54	85.08	65.86	0.41	5.58	3.60	31.26
	*Lobophora variegata*	–	–	4.22	–	–	2.23	–	–	–	–	–	0.59
	*Padina crassa*	–	–	3.45	–	–	–	0.77	0.61	–	6.12	–	0.99
	*Padina japonica*	–	–	0.86	–	–	–	–	–	–	–	–	0.08
	*Zonaria diesingiana*	–	–	6.23	–	–	–	–	–	–	–	–	0.57
Ectocarpaceae	*Bodanella lauterborni*	–	–	–	–	–	–	–	0.12	–	–	–	0.01
	*Ectocarpus* *crouaniorum*	–	100.00	–	–	–	–	–	–	5.31	60.07	64.00	20.85
	*Myagropsis myagroides*	–	–	2.01	1.17	10.96	–	–	20.06	–	–	–	3.11
Sargassaceae	*Phaeostrophion* *irregulare*	–	–	0.77	–	–	–	–	–	–	–	–	0.07
	*Sargassum muticum*	51.98	–	11.79	98.32	87.38	5.17	11.83	10.28	92.96	3.96	13.20	35.17

**Notes:**

^a–c^The same letter indicates a pair for mating.

* Seaweeds in gut contents.

### Carbon and nitrogen stable isotope ratio analysis

**Figure 9:**
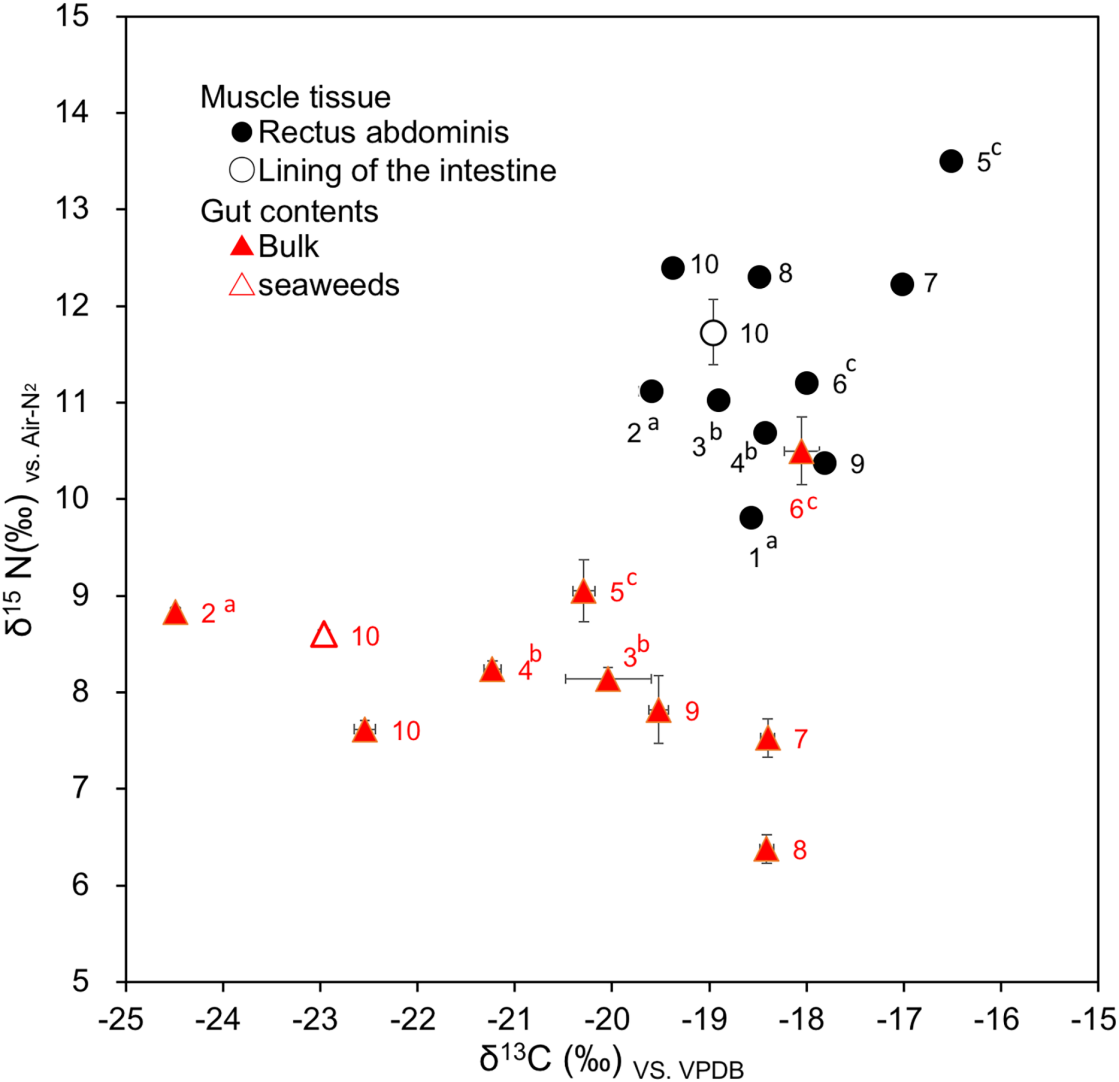
Relationship between carbon and nitrogen stable isotope ratios in muscle tissues (rectus abdominis and lining of the intestine) and gut contents of green turtles, *Chelonia mydas*, collected from Hahajima I., Ogasawara Is., Japan. Numbers indicate the turtle number. ^a–c^The same letter indicates a pair for mating.

In the green turtles’ muscle tissue (rectus abdominis), the carbon stable isotope ratio ranged from −19.60‰ to −16.52‰, while the nitrogen stable isotope ratio ranged from 9.81‰ to 13.51‰. Similarly, in the gut contents, the carbon stable isotope ratio ranged from −24.49‰ to −18.05‰, while the nitrogen stable isotope ratio ranged from 6.38‰ to 10.50‰ (Table 6, Fig. 9). Thus, both the carbon and nitrogen stable isotope ratios of the muscle tissue and the gut contents collected from the same individual tended to be higher in the muscle tissue than in the gut contents. Namely, the average carbon stable isotope ratio of the muscle tissue (−18.24‰ ± 1.02‰, *n* = 9) was higher than that of the gut contents (−20.33 ± 2.13‰, *n* = 9), and the difference between them was significant (*t*-test, *t*(8) = 3.72, *p* < 0.01). The average nitrogen stable isotope ratio of muscle tissue (11.65 ± 1.01‰, *n* = 9) was higher than that of the gut contents (8.23 ± 1.15‰, *n* = 9), and this difference was also significant (*t*-test, *t*(8) = 6.26, *p* < 0.001). The average difference between the gut contents and muscle tissue was 2.09‰ for the carbon stable isotope ratio and 3.42‰ for the nitrogen stable isotope ratio.

**Table 6:** Carbon and nitrogen stable isotope ratios of green turtles, *Chelonia mydas*, collected from Hahajima I., Ogasawara Is., Japan.

Turtle number	Sex	Muscle tissue (rectus abdominis)		Gut contents		Difference between tissue and gut contents	
		Carbon (‰)	Nitrogen (‰)	Carbon (‰)	Nitrogen (‰)	Carbon (‰)	Nitrogen (‰)
1^a^	M	−18.57 ± 0.10	9.81 ± 0.10	–	–		
2^a^	F	−19.60 ± 0.12	11.12 ± 0.02	−24.49 ± 0.05	8.83 ± 0.05	4.89	2.29
3^b^	M	−18.91 ± 0.07	11.03 ± 0.07	−20.04 ± 0.44	8.14 ± 0.12	1.13	2.89
4^b^	F	−18.43 ± 0.06	10.69 ± 0.02	−21.23 ± 0.09	8.24 ± 0.09	2.80	2.45
5^c^	M	−16.52 ± 0.11	13.51 ± 0.10	−20.29 ± 0.11	9.05 ± 0.32	3.77	4.46
6^c^	F	−18.00 ± 0.05	11.21 ± 0.08	−18.05 ± 0.18	10.50 ± 0.35	0.05	0.71
7	M	−17.02 ± 0.11	12.23 ± 0.05	−18.40 ± 0.07	7.53 ± 0.20	1.38	4.70
8	M	−18.49 ± 0.05	12.31 ± 0.08	−18.41 ± 0.07	6.38 ± 0.15	−0.08	5.93
9	F	−17.82 ± 0.12	10.38 ± 0.07	−19.52 ± 0.10	7.82 ± 0.35	1.70	2.56
10-1	M	−19.38 ± 0.07*	12.40 ± 0.03*	−22.54 ± 0.11**	7.62 ± 0.09**	3.16	4.78
10-2		−18.96 ± 0.03*	11.73 ± 0.34*	−22.96 ± 0.06**	8.60 ± 0.02**		

**Notes:**

M, male; F, female.

^a–c^The same letter indicates a pair for mating.

* For individual 10, the rectus abdominis (10-1) and the lining of the intestine (10-2) were analysed. There were significant differences between them in a *t*-test: carbon, *t*(4) = 7.90, *p* < 0.01; nitrogen, *t*(4) = 4.59, *p* < 0.05.

** For individual 10, the bulk of the gut contents (10-1) and the seaweed (10-2) were analysed. There were significant differences between them in a *t*-test: carbon, *t*(4) = 4.59, *p* < 0.05; nitrogen, *t*(4) = 14.20, *p* < 0.001.

Comparing males and females, the average carbon stable isotope ratio was −17.91 ± 0.95‰ (*n* = 6) for males and −18.46 ± 0.80‰ (*n* = 4) for females, with no significant difference between males and females (*t*-test, *t*(8) = 0.95, *p* = 0.37). The average nitrogen stable isotope ratio was 11.88 ± 1.28‰ (*n* = 6) for males and 10.85 ± 0.39‰ (*n* = 4) for females, with no significant difference between them (*t*-test, *t*(8) = 1.53, *p* = 0.16). As for the mating pairs, Table 7 shows how the carbon and nitrogen stable isotope ratios of the muscle tissue and gut contents differed between the individuals of each pair. Apart from an insignificant difference of 0.1‰ in the nitrogen stable isotope ratios of the gut contents of turtles no. 3 and 4 of pair B, significant individual differences, ranging from 0.34‰ to 2.30‰, were found in all the pairs.

**Table 7:** Differences in carbon and nitrogen stable isotope ratios between individuals of green turtle, *Chelonia mydas*, comprising a pair for mating.

Pair	Turtle number		Muscle tissue						Gut contents					
	Male	Female	Carbon (‰)	*t* value	*p* value	Nitrogen (‰)	*t* value	*p* value	Carbon (‰)	*t* value	*p* value	Nitrogen (‰)	*t* value	*p* value
A	1	2	1.02	9.33	<0.001	1.31	−18.27	<0.001	–	–	–	–	–	–
B	3	4	0.48	−7.90	<0.01	0.34	6.73	<0.01	1.19	3.75	<0.05	0.10	−0.94	0.40
C	5	6	1.48	16.88	<0.001	2.30	26.09	<0.001	2.20	−15.96	<0.001	1.37	−4.86	<0.001

For turtle no. 10, in addition to muscle tissue and gut contents, we analysed the lining of the intestine and seaweed taken from the gut contents. Significant differences between the rectus abdominis and the lining of the intestine were observed in the carbon as well as the nitrogen stable isotope ratios, both within 0.7‰ (δ^13^C: difference = 0.42, *t-*test, *t*(4) = 7.80, *p* < 0.01. δ^15^N: difference = 0.67, *t-*test, *t*(4) = 2.78, *p* < 0.05). Similarly, between the bulk of the gut contents and the seaweed, the carbon and nitrogen stable isotope ratios differed significantly, both within 1.0‰ (δ^13^C: difference = 0.42, *t-*test, *t*(4) = 4.56, *p* < 0.05. δ^15^N: difference = 0.98, *t-*test, *t*(4) = 14.20, *p* < 0.001) (Table 6).

The green turtles were divided into three groups according to the dominant seaweeds identified in the genetic analysis of the gut contents mentioned above (Fig. 8). The carbon stable isotope ratio of the gut contents differed significantly between the three groups (one-way ANOVA, *F*(2, 7) = 21.49, *p* < 0.01). However, neither the nitrogen stable isotope ratio of the gut contents (one-way ANOVA, *F*(2, 7) = 0.04, *p* = 0.96), nor the carbon stable isotope ratio of the muscle tissue (one-way ANOVA, *F*(2, 7) = 4.05, *p* = 0.07), nor the nitrogen stable isotope ratio of the muscle tissue (one-way ANOVA, *F*(2, 7) = 0.04, *p* = 0.96) were significantly different.

## Discussion

### Food habitat and ingestion of plastics

In this study, micro-, meso- and macroplastics were found in seven of the 10 green turtles examined. Meso- and macroplastics were found in six of the 10 turtles, with an average of 9.2 ± 11.48 items/individual (range: 0–31, *n* = 10). The possession rate of plastic items has been reported to be 0–88% in green turtles from the Pacific, Atlantic, Indian, and Mediterranean Oceans and 70% in the Northwest Pacific (Lynch, 2018). In green turtles from the coast of Tohoku, Japan, Fukuoka et al. (2016) reported a possession rate of 84.6% (11 out of 13 individuals), which was similar to the rates reported in previous studies. In the Mediterranean, the rate is low, at less than 5% (Lynch, 2018), but in green turtles from Cyprus in the eastern Mediterranean, all 19 individuals examined had ingested plastic (Duncan et al., 2019a). However, Duncan et al. (2019a) counted plastics in the entire digestive tract, including the esophagus, stomach, and intestines, so the possession rate was likely to be high. The possession rate of 70% found in this study was based on collecting plastics only from the large intestine, so it is not possible to make a general comparison with studies (Lynch, 2018; Duncan et al., 2019a) that examined the number and weight of all ingested plastics per individual. Taking this limitation into account, however, when compared with the data summarised in Lynch (2018), while the average number of pieces per individual found in this study (9.2 items/individual) was lower than that reported by Lynch (2018) (approximately 70 items/individual), the weight of the plastic in our case (15.28 g/individual) was higher than the maximum value (just under 10 g) in Lynch (2018), indicating that the plastic items ingested by the green turtles in this study were heavier, *i.e*., larger, than those reported by Lynch (2018).

In the present study, seaweed accounted for 97.3% (average value, excluding non-organic matter, by weight %) of the contents of the large intestine of the 10 adult green turtles that were the subject of this study (Table 3), indicating that they were herbivorous. This is consistent with previous findings that juvenile green turtles are omnivorous but adults are herbivorous, feeding on seaweed and sea grass as their main food source (Brand-Gardner, Limpus & Lanyon, 1999; Kameda & Isihara, 2009; Schuyler et al., 2012; Fukuoka et al., 2016; Duncan et al., 2019a). The results of genetic analysis showed that brown algae such as Dictyotaceae, Sargassaceae and Ectocarpaceae accounted for 95.38% of the total number of seaweed reads in the gut contents (Table 5, Fig. 8). The seaweed vegetation of the Ogasawara Islands is tropical, with a high proportion of green algae and a low proportion of brown algae (Ohba et al., 1998; Yabe, Ishii & Nohara, 2022), so there is a discrepancy between the seaweed composition in the gut contents and the seaweed vegetation of the Ogasawara Islands. Although no quantitative information has been reported on seaweeds in the Ogasawara Islands, according to Inaba (2004) the amount of *Sargassum duplicatum* and *Dictyota spinulosa* has increased in recent years. However, no DNA of those two species was detected in the current survey (Table 5). The limited knowledge of the seaweed flora in the Ogasawara Islands makes it difficult to determine whether the green turtles in the Ogasawara Islands are selective feeders, seeking out the limited number of brown algae species, or opportunistic feeders, feeding non-selectively on brown algae when the supply increases (Hirai, Kameda & Kawamura, 2020). A quantitative study of seaweed flora along the Ogasawara coast is needed to examine this issue. In addition, in the seas around Japan, *Sargassum* species account for most of the floating seaweeds. *Sargassum* species have vesicles that can store gas, enabling them to float, and their ability to grow while drifting enables them to travel long distances (Komatsu et al., 2009; Rothäusler, Gutow & Thiel, 2012). It is therefore possible that the individuals that fed on *Sargassum* species were feeding on drifting seaweed originating from the Ogasawara Islands or elsewhere. In this study, the total number of reads for all life forms in the genetic analysis of the gut contents of the green turtles was dominated by the phyla Platyhelminthes (flatworms), Bacillariophyta (diatoms), Alveolata (protozoa) and Phaeophyceae (brown algae) (Fig. 6); and 80% of the reads derived from Metazoa were benthic or epiphytic organisms (Table 4), which live on seaweed leaves and are thought to have been ingested along with the seaweed (Kito, 1975; Thiel & Gutow, 2005).

As mentioned above, it has been established that green turtles in the seas around the Ogasawara Islands are herbivorous and that many of them ingest plastic, but it is not fully understood why they do this (Schuyler et al., 2012). According to this study, green turtles may be ingesting plastic for two main reasons: (1) they are accidentally ingesting plastic mixed in with their main food source of seaweed and (2) they are selectively ingesting plastic, mistaking it for food (Fukuoka et al., 2016). Regarding reason 1, Li et al. (2022) investigated the plastic found among five species of large algae distributed in the coastal areas of China and showed that all sizes of plastic, including macro-, meso-, and microplastics, were present in abundances of 0–201.5, 0–1,178.0, and 0–355.6 items/kg seaweed dry weight, respectively. The Ogasawara Islands are geographically separated from the coast of China where Li et al. (2022) conducted their study, and their seaweed vegetation is also considered to be different. However, assuming that this is a general trend for plastics to abound among large seaweeds, it is highly possible that green turtles eat plastics opportunistically along with seaweed (Di Beneditto & Awabdi, 2014). On the other hand, Duncan et al. (2019a) showed that, compared to plastic beach debris, green turtles (*Chelonia mydas*) that feed primarily on sea grass in the eastern Mediterranean Sea preferentially ingest certain types of plastic that resemble sea grass in shape and colour. No information is available on plastics present in the seaweed beds where green turtles living in the waters around the Ogasawara Islands feed. So, assuming the data in Li et al. (2022) reflects the norm, we compared the plastics present in the seaweed beds they studied with those ingested by the green turtles in the present study. In terms of shape, Li et al. (2022) reported that fibres (52.2%) accounted for most of the plastics found among the seaweeds, while 1–5 mm (39.6%) dominated in terms of size, and polystyrene (36.5%) in terms of material. In contrast, the ingested plastics retrieved in the present study were predominantly film and fragments, accounting for 44.6% and 42.4%, respectively, and the proportion of lines, which corresponds to fibres, was low at 13.0%. The sizes were mostly ≥1 cm^2^ (Fig. 1), and 79.5% of the material was polyethylene, with polystyrene accounting for only 4.5%. Moreover, 19.6% of the ingested plastics were black and 29.0% were coloured. Based on the disparity between the results reported by Li et al. (2022) and the findings of the present study, it is conceivable that green turtles selectively feed on relatively large, film-like plastics resembling seaweed. To clarify whether herbivorous green turtles ingest plastic selectively as opposed to accidentally, further investigation of the plastic present in seaweed beds and among floating seaweeds is required.

As for reason 2 above, previous studies have suggested that sea turtles may ingest plastic on purpose, mistaking it for food (Mrosovsky, Ryan & James, 2009; Schuyler et al., 2012; Duncan et al., 2019a). As previously mentioned, green turtles in the eastern Mediterranean Sea have been shown to preferentially ingest plastics that resemble sea grass, their principal food (Duncan et al., 2019a). Jellyfish have also been widely discussed, and there is a strong argument for the hypothesis that sea turtles feed selectively on plastic, mistaking it for jellyfish, one of their normal foods (Schuyler et al., 2014). Fukuoka et al. (2016) observed that green and loggerhead sea turtles fitted with video cameras visually confused transparent, drifting soft-plastic debris with gelatinous prey such as jellyfish and salpas. Furthermore, the encounter-ingestion ratio of artificial debris was significantly higher in the green turtles than the loggerhead turtles, indicating that green turtles are attracted to artificial objects. In the present study, the results of genetic analysis of the gut contents showed that gelatinous plankton accounted for 17.67% of the metazoans, and they appeared in all individuals (Fig. 7). Turtles no. 4 and 5 had a particularly high proportion of these, accounting for 50.03% and 51.15%, respectively. Moreover, nine of the 26 plastic items ingested by turtle no. 4 and six of the nine plastic items ingested by turtle no. 5 were clear, translucent or white (Fig. 4), *i.e*., similar in colour to gelatinous plankton. Combined with the fact that both individuals were feeding mainly on the drifting seaweed *Sargassum muticum*, these findings suggest that turtles no. 4 and 5 were feeding on gelatinous plankton in the surface layer while also feeding on plastic by mistake. To determine whether green turtles are selectively ingesting plastics, quantitative data on macro- and megaplastics in the marine environment are severely lacking. Future quantitative studies are required on the quantity, size distribution, shape, and colour of plastics distributed in surface waters, seagrass beds and drifting seaweeds in the distribution areas of green turtles. These data need to be compared with macro- and megaplastics ingested by green turtles to examine the selectivity of their plastic ingestion.

Regarding seasonal variation in plastic ingestion, it is known that geophysical features such as current direction, precipitation and human activities are contributing factors. For example, higher concentrations of floating microplastics and microplastics in mangrove sediments are reported to exist in the wet season than the dry season (*e.g*., Garcés-Ordóñez et al., 2023; Nakano et al., 2024), and microplastics accumulate, for example, due to the convergence of currents (in the vicinity of a thermohaline front) (Nakano, Arakawa & Tokai, 2021). Also, more abundant quantities of plastic litter were found on a sandy beach in a specific season (*e.g*., Burlat & Thorsteinsson, 2022), and simulated results showed that seasonality such as this would depend on the activities of local fisheries (Rieger et al., 2024). If plastic abundance in the environment is seasonal, as described above, it follows that the extent of plastic ingestion is also subject to seasonal variation. Nevertheless, seasonality has not been discussed based on the data we collected due to the single sampling period (March 2021) and small sample size of this study. To capture seasonal variations in plastic ingestion, further studies across multiple seasons are warranted. In addition, green turtles may ingest plastics during migration, so we need to consider their ingestion pattern not only around the Ogasawara Islands but in the different sea areas through which they migrate. However, it is difficult to find migrating green turtles and obtain data on plastic ingestion without harming the turtles, so samples are rarely collected and data is limited. This kind of analysis is challenging all over the world.

Some of the plastic items retrieved from the green turtles in this study were imprinted with simplified or traditional Chinese characters, Japanese characters or letters from the Korean or Roman alphabets (Fig. 5). While these characters do not directly indicate the origin of the plastics, they may at least indicate the trading area of the products. The estimated trading area of China, Taiwan, the Korean Peninsula, and Japan is wider than the distribution and migration range of green turtles from the Pacific coast of Japan to the Ogasawara Islands. It is conceivable that plastics discharged into the ocean from the coasts of those commercial areas, as well as from offshore industries such as shipping and fishing and from ship-waste dumping, flowed into the green turtles’ habitat, where they were ingested by the turtles. This study showed that green turtles accumulate plastics from a wide range of origins, constituting a typical example of transboundary pollution.

### Carbon and nitrogen stable isotope ratios and sea turtle migration

In the predator-prey relationship, it is generally known that the carbon stable isotope ratios of prey and predator are close, and the nitrogen stable isotope ratio of a predator is 3.4‰ higher than that of its prey (DeNiro & Epstein, 1978; Minagawa & Wada, 1984; Wada et al., 1987). In this study, the carbon and nitrogen stable isotope ratios of the muscle tissue (rectus abdominis) and gut contents collected from the same individual tended to be lower in the gut contents than in the muscle tissue (Fig. 9), indicating that the diet is basically reflected in the muscle tissue. However, the difference in the nitrogen stable isotope ratios between the gut contents and muscle tissue of each individual ranged from 0.71‰ to 5.93‰ (Table 6), which differs from the common value of 3.4‰ for most individuals. This suggests that the muscle tissue reflected not the current diet, but variable feeding histories. Estimating the feeding history of each individual, the difference between the carbon stable isotope ratios of the gut contents and muscle tissue of turtles no. 2, 5, and 10 was large, at 3.16–4.89‰ (Table 6), so it is possible that they were previously eating food with higher carbon stable isotope ratios than their current diet. In addition, compared to the other individuals, the difference between the nitrogen stable isotope ratios of the gut contents and muscle tissue of turtles no. 5, 7, 8, and 10 was relatively high, at 4.46–5.93‰, indicating that they may have been eating food with higher nitrogen stable isotope ratios in the past than when they were captured. For turtles no. 7 and 8, the carbon stable isotope ratio in the muscle tissue was the same as in the gut content, but the nitrogen stable isotope ratio in the muscle tissue was high, so it is likely that their past diet also differed from their most recent one.

The DNA analysis results suggest that the feeding grounds at the time of capture were three locations where the seaweeds *Ectocarpus crouaniorum*, *Sargassum muticum*, and *Lobophora* sp. dominate (Fig. 8). The carbon stable isotope ratios of the muscle tissue ranged from −19.60‰ to −16.52‰ (Fig. 9), indicating that *Ectocarpus crouaniorum* and Rhodophyta, which have lower values than this range, were not eaten in the past, while *Sargassum muticum*, *Lobophora* sp., and Chlolophyta were. The carbon and nitrogen stable isotope ratios in the muscle tissue of turtles no. 5 and 7 were both high, with carbon stable isotope ratios of −16.52‰ and −17.02‰, and nitrogen stable isotope ratios of 13.51‰ and 12.23‰, respectively (Fig. 9). These values are close to the carbon and nitrogen stable isotope ratios of loggerhead sea turtles that feed on jellyfish in the seas around Japan (Hatase et al., 2002; Ishihara, 2012), so it is possible that turtles no. 5 and 7 fed mainly on plankton, including jellyfish, while migrating. Green turtles migrate southward from the Pacific coasts of the Japanese archipelago to the Ogasawara Islands for mating and nesting, and the young turtles hatched in the Ogasawara Islands grow up and migrate to the coasts of the Japanese archipelago (Kamezaki & Matsui, 1977; Ogasawara Marine Center, 1990; Tachikawa & Sasaki, 1990; Tachikawa, 1991; Yamaguchi, Suganuma & Narushima, 2005). The results of this study suggest that these green turtles were essentially herbivorous but, after leaving the coast on their way to the Ogasawara Islands, they fed on not only drifting seaweeds consisting of *Sargassum* but also gelatinous plankton such as jellyfish and salpas, raising the possibility that they may have temporarily adopted a carnivorous or omnivorous feeding mode during their southward migration. This hypothesis is supported by previous studies. It has been reported that green turtles in the seas around Japan, which feed mainly on seaweeds, also feed on gelatinous plankton such as jellyfish (Hatase et al., 2006; Fukuoka, Narazaki & Sato, 2015; Fukuoka et al., 2019). A study of the foraging habitat of green turtles on the Sanriku Coast of northern Japan using biologging experiments and carbon and nitrogen stable isotope ratio analysis (Fukuoka et al., 2019) showed that the Sanriku Coast is a summer feeding ground where the turtles feed exclusively on macroalgae, after which they obtain relatively high energy compared to other sea areas by feeding on gelatinous food as they make their way southward for the winter.

Comparing the carbon and nitrogen stable isotope ratios in the muscle tissue of the male and female of each pair, a large difference was observed between the male and female of pairs A and C (turtles no. 1–2 and 5–6). In pair C, there was also a large difference in the carbon and nitrogen stable isotope ratios of the brown algae in the gut contents, and the DNA composition of the brown algae also differed. In addition, although the carbon and nitrogen stable isotope ratios in the muscle tissue and the carbon stable isotope ratio in the gut contents of pair B (turtles no. 3 and 4) differed only slightly, the DNA composition of the brown algae was different (Table 7). These findings indicate that the behavioural history of the males and females of each pair differed before they met, as did their migratory destination and the length of time they spent elsewhere before coming to the waters near Hahajima Island and forming pairs. For sea turtles, the turnover time for the isotope ratio of food to be reflected in the muscle tissue is unknown, but the plasma reflects feeding close to the time of collection, and the epidermis from several months before (González Carman et al., 2013; Vander Zanden et al., 2014). It is therefore likely that the males and females were feeding in different locations several months before they met and formed pairs. Furthermore, the differences between them in the DNA composition of brown algae, their main food source, suggest that the pairs had not been together long.

## Conclusion

This research confirmed through gut-content analysis that adult green turtles feed mainly on seaweeds and showed that they may ingest meso- and macroplastics entangled in seaweed beds and drifting seaweeds when feeding. Furthermore, the research established through DNA analysis that green turtles feed on gelatinous plankton such as jellyfish and salpas. To determine whether green turtles feed selectively on plastics, we would need to compare the percentages of plastics of various shapes, colours and sizes present in the environment with those ingested. Since no such information concerning the marine environment was available to us for this study, we cannot comment conclusively on the selectivity of the green turtles’ plastic ingestion. However, based on various data, including DNA analysis and the fact that a large proportion of the ingested plastics were white or semi-transparent, we have demonstrated the possibility that green turtles feed selectively on plastics, mistaking them for jellyfish. Although the sample size was small and the sampling period limited, the use of multiple analytical methods enabled us to draw the above conclusions, and the dual sampling of turtle no. 10 validated consistency, enhancing the robustness of our findings. To obtain more accurate results, the sample size would need to be increased and the sampling process conducted throughout the year. However, it is difficult to collect samples systematically from green turtles migrating in the open ocean. Future research should therefore focus on developing and refining survey methods, such as collecting faeces from migrating green turtles and using these for isotopic, DNA and plastic analyses, as well as conducting biologging experiments.

Plastic ingestion may have repercussions for green turtles, including tissue damage resulting from physical injury, reduced food intake, and effects on excretion. Indeed, in this study, mottled black discolouration was observed on the intestinal lining of the individual that had ingested the greatest number and volume of plastic items and had the highest proportion of plastic weight to body weight. While no histological studies have been conducted and the causal relationship with the number and volume of plastics ingested is unknown, this black discolouration suggests an effect of plastic ingestion on the organism. Regardless of the quantity ingested, previous studies have pointed out that even one piece of plastic can cause serious damage (Bjorndal, Bolten & Lagueux, 1994; Wilcox et al., 2018). The toxic compounds contained in plastics may also be detrimental (Yamashita et al., 2011; Tanaka et al., 2013; Nelms et al., 2016; De-la-Torre et al., 2021; Fukuoka et al., 2022). In fact, chemical substances such as PCBs and PAHs, which are found in plastics, have been detected in a variety of wild animals including seabirds (Navarro et al., 2023) and finless porpoises (Xie et al., 2024), and exposure to plastic additives such as these in laboratory experiments has been shown to impact the behaviour and health of species such as marine amphipods (Green-Ojo et al., 2024a, 2024b) and zebrafish (Qualhato, Dias & Rocha, 2024). Although there are discrepancies between the levels of exposure in the laboratory and contamination in the environment, plastic pollution could potentially affect the health of organisms. While plastic additives were not examined in this study, if plastics are chemically toxic, it has been pointed out that large plastics are more likely to be harmful, in which case sea turtles, which ingest large plastics, will be more seriously affected (Lim, 2021). Moving forward, it will be necessary to identify the additives in plastic waste and clarify their effects on sea turtles and other marine organisms.

By confirming that green turtles ingest meso- and macroplastics from transboundary sources, likely mistaking them for gelatinous plankton, this study has filled the gap in understanding migration-related ingestion in the Ogasawara Islands. Moreover, by demonstrating that plastic pollution is a transnational problem, our integrated analyses have illustrated the essential value of collecting data on green turtles and other migratory marine species, even incidentally, within an international framework. Cooperative data compilation could contribute to not only understanding the effects of plastic pollution but also conserving green turtles and other species that migrate around the world. Furthermore, ongoing research should be accompanied by international collaboration to reduce plastic pollution.

## Supplemental Information

10.7717/peerj.20425/supp-1 Supplemental Information 1 Properties of plastics in gut contents of of adult green turtles, *Chelonia mydas*, in Hahajima I., Ogasawara Islands, Japan.

10.7717/peerj.20425/supp-2 Supplemental Information 2 The number of reads for eukaryote excluding sea turtles and intestinal parasites in gut contents of of adult green turtles, *Chelonia mydas*, in Hahajima I., Ogasawara Islands, Japan.

## Additional Information and Declarations

### Field Study Permissions

The following information was supplied relating to field study approvals (*i.e*., approving body and any reference numbers):

The head of the Ogasawara Hahajima Fisheries Cooperative.

### DNA Deposition

The following information was supplied regarding the deposition of DNA sequences:

The FASTQ files are available at the DNA Data Bank of Japan (DDBJ): DRA020537 and NCBI: PRJDB20659.

### Data Availability

The following information was supplied regarding data availability:

The raw measurements are available in the Supplemental Files.
